# A catalogue of virulence strategies mediated by phytopathogenic effectors

**DOI:** 10.1016/j.fmre.2023.10.026

**Published:** 2024-02-21

**Authors:** Gan Ai, Hao Peng, Weiye Pan, Yuke Li, Zhirui Wan, Zhiyuan Yin, Danyu Shen, Suomeng Dong, Yuanchao Wang, Daolong Dou

**Affiliations:** a College of Plant Protection, Academy for Advanced Interdisciplinary Studies, Nanjing Agricultural University, Nanjing 210095, China; b USDA-ARS, Crop Diseases, Pests and Genetics Research Unit, Parlier, CA 93648, USA

**Keywords:** Plant pathogenic organism, Effector, Host immunity, Pathogen-host interaction, Effector action mode

## Abstract

Plant diseases cause dramatic economic loss, posing a major challenge to modern agriculture. Plant pathogenic organisms secret effectors that utilize fascinating and intricate stratagems to facilitate infection. The consequences of plant-pathogen interactions are largely determined by effectors. The effector research has made great strides since its inception in the 1990s and the importance of effectors is increasingly noticed. Molecular investigation of effectors has provided critical insights into how plant pathogens manipulate their hosts to cause diseases. Thus far, numerous excellent reviews concerning effectors have focused on their targeting host pathways, recognition by host receptors, and evasion mechanisms, but few have ever summarized all known effector action modes. Here, we distinguish ten different stratagems of effector function from all types of pathogens, including damage, inhibition, hijacking, promotion, subversion, mimicry, reprogramming, evasion, decoying, and adaption. Furthermore, we discuss examples of these ten stratagems, refine the effector definition, and propose future directions of phytopathogenic effector research.

## 1 Introduction

**Fig. 1:**
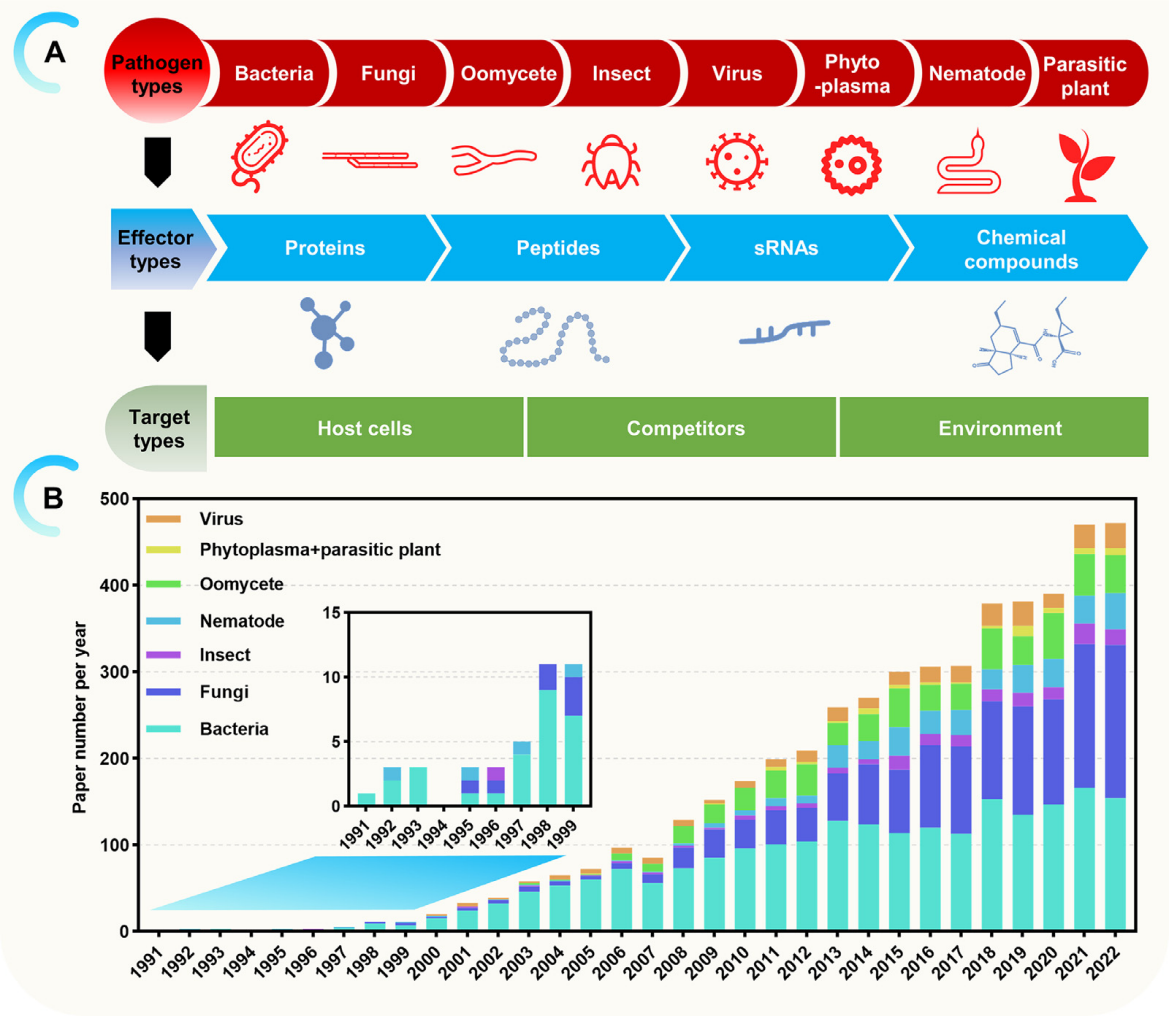
**A bird's-eye view of effectors in plant pathogenic organisms**. (A) Effector characterization from plant pathogenic organisms. Known types of pathogens, effectors and their targets are shown. (B) A historic overview of effector studies. The number of pathogenic effector-related papers per year is shown. Data were obtained from Web of Science (https://www.webofscience.com/wos/alldb/basic-search).

Global food security faces tremendous pressures from climate change and population growth. Moreover, crop diseases continue to exert a significant negative economic impact [1]. Fungal and bacterial pathogens are estimated to reduce crop yields by 15% on average, while viral pathogens lead to about 3% loss [2]. The impact is particularly devastating for certain crops. For example, microbial infection causes nearly 30% reduction in potato yield [2]. Plant diseases are caused by a wide range of pathogenic organisms with different infection strategies. Known plant pathogens include bacteria, fungi, insects, oomycetes, viruses, phytoplasmas, and parasitic plants. They invade different host tissues and have evolved diverse mechanisms to overcome plant defenses (Fig. 1A). To defend against pathogens, plants have developed an immune system that recognizes microbial invaders and activates defense mechanisms to hamper their proliferation. In contrast, pathogens also have their own weapons, which are secreted effectors. Many effectors manipulate plant immunity pathway or create a favorable environment to facilitate pathogen colonization, while some are also recognized by plants, thus attenuating the affinity between pathogens and plants (Fig. 1A) [3]. Importantly, the consequences of plant-pathogens interactions are largely determined by effectors.

Effector research in plants first focused on the avirulent activity of effectors from phytopathogenic bacteria (Fig. 1B). In the early 2000s, research on effectors continued to expand to more pathogens (Fig. 1B). Nowadays, our understanding on effectors is more comprehensive. There are numerous excellent reviews focusing on effector-targeted host pathways, as well as effector recognition by host receptors and evasion from plant immunity [4,^3^,5]. However, few reviews focus on the mode of action used by pathogenic effectors to manipulate pathogen-host infections. In this review, we summarize ten action stratagems used by effectors to function, giving examples for each stratagem.

## 2 Definition of effectors

We propose the following definition: an effector is a pathogen-secreted molecule that functions to induce non-self-phenotypes in other organisms or the environment. Below we explain the two fundamental criteria of this revised definition and how it addresses the shortcomings of previous definitions commonly adopted in the field.

### 2.1 Molecule

The prevalent definitions have often confined effectors to proteins, which undisputedly represent the most abundant effector category identified thus far. However, the identification and functional elucidation of other effector types challenge the conventional definitions. Presently, effectors can encompass small RNAs, peptides, or non-protein chemical compounds (Fig. 1A).

Small RNA-type effectors were initially identified in the necrotrophic fungus *Botrytis cinerea*. During early infection, small RNA-type effectors move into host plant cells and silence important immunity genes [6]. Subsequent discoveries uncovered small RNA effectors in other fungi such as *Verticillium dahlia* and *Puccinia striiformis*, as well as in bacteria and parasitic plants [7].

Peptides represent another prevalent effector category. A famous example is the rapid alkalinization factor (RALF)-like peptides secreted by the root-infecting fungus *Fusarium oxysporum* (F-RALFs). F-RALF effectors can mimic plant RALF peptides to get recognized by plant cells and induce extracellular alkalinization, which is important for *F. oxysporum* pathogenesis in plants [8]. CLAVATA3/EMBRYO SURROUNDING REGION-RELATED (CLE) peptide effectors from nematode are another large peptide effector family. Nematode CLEs mimic plant CLE peptides to bind to membrane-located receptors and manipulate plant cell differentiation [9]. Other famous peptides, such as flagellin 22 (flg22), translation elongation factor Tu (elf18), and necrosis and ethylene-inducing peptide 1-like 20 (nlp20), are pathogen-associated molecular patterns (PAMPs) that induce plant defense pathways [3].

Chemical compounds belong to another type of pathogen armament. Some bioactive metabolites may act as effectors to suppress plant immunity. For instance, the bacterial pathogen *Pseudomonas syringae* produces coronatine, an chemical effector that simulates an active form of jasmonic acid (JA) to repress the antagonistic salicylic acid (SA)-mediated immune response via cross-communication between the two pathways [10]. Other small molecule compounds might be toxins that kill plant cells. The mycotoxin fumonisin from *Fusarium* fungi not only damages plant cells but also poses a threat to food safety and public health by contaminating various agricultural commodities, incurring deleterious effects on both human and livestock consumers [11].

### 2.2 Non-self-phenotype

Apart from the widely used definitions of effector functioning in regulating plant immunity, effectors may also target other species in the same niche or directly change environment conditions to facilitate colonization. For example, some pathogens deploy their effectors to reshape host microbiomes for their own advantages. The *Verticillium dahliae* virulence effector VdAve1 manipulates tomato and cotton microbiomes by suppressing antagonistic bacteria [12]. In addition, effectors can generate a suitable environment for pathogenesis. *Blumeria graminis* f.sp. *hordei* (Bgh), an obligate biotrophic fungal pathogen of barley, elicits a H_2_O_2_ burst at the germ tube invasion sites. To counteract this defense response, Bgh secrets an effector named catalase B, to detoxify H_2_O_2_ and thereby promote infection [13].

## 3 The ten action stratagems of phytopathogenic effectors

**Fig. 2:**
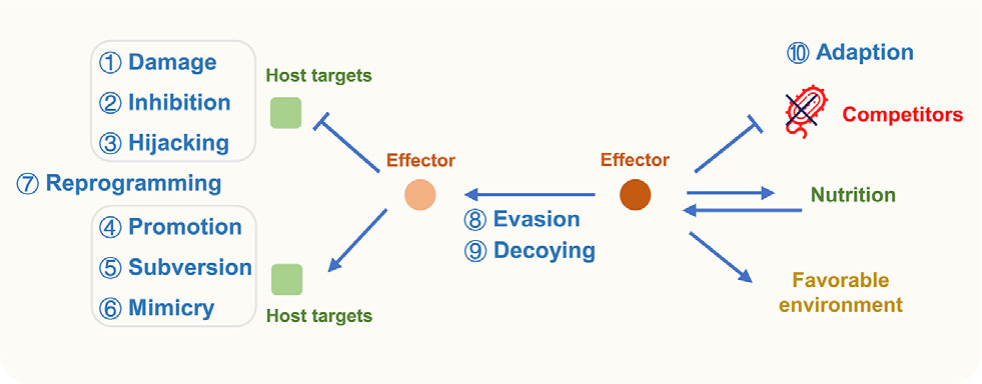
**Summarization of the ten action stratagems of phytopathogenic effectors**. The ten stratagems are damage, inhibition, hijacking, promotion, subversion, mimicry, reprogramming, evasion, decoying, and adaption. The stratagems of the effectors are written in blue, with an upward arrow denoting increase and a downward arrow denoting decrease. Effectors can interfere with target functions through damage, inhibition, and hijacking stratagems. They also promote target functions through promotion, subversion, and mimicry stratagems. Meanwhile, they could also manipulate plant immunity by the reprogramming stratagem. Effectors may also prevent recognition of another effector by host through evasion and decoying stratagems. Additionally, effectors can target other species in the same niche, directly change environment conditions to facilitate colonization, or acquire nutrients through the adaption stratagem.

In this review, we tease the ten effector action stratagems based on current knowledge. In general, effector modes of action can be classified into 10 types, including damage, inhibition, hijacking, promotion, subversion, mimicry, reprogramming, evasion, decoying, and adaption (Fig. 2).

### 3.1 Stratagem 1: damage

**Fig. 3:**
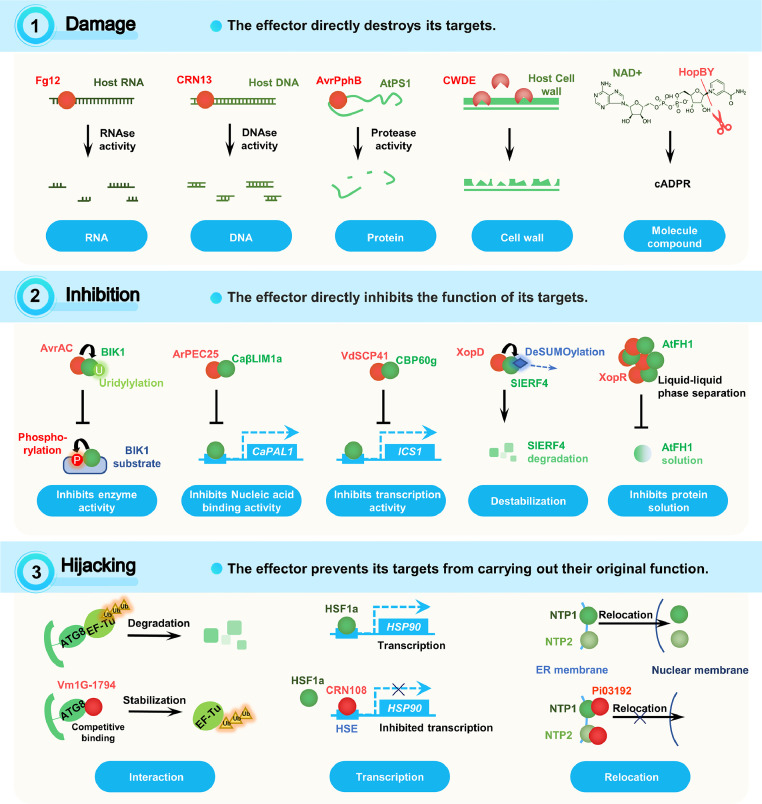
**Plant targets and effector stratagems: damage, inhibition, and hijacking**. The description of the indicated stratagems and the illustration of examples for these stratagems are shown. Effectors are shown in red. The targets of effectors are shown in green. The stratagems of the effectors are written above, with an upward arrow denoting increase and a downward arrow denoting decrease. The detailed target categories of the indicated effectors are written below.

Damage refers to the direct destruction of targets by effectors. Known destroyed targets include host proteins, RNAs, DNAs, cell walls, membranes, and other molecular components (Fig. 3).

#### 3.1.1 Host proteins

Host proteins are common targets of effector-mediated damage. *P. syringae* type III effector (T3E) AvrPphB proteolytically cleaves its targeting kinase AVRPPHB SUSCEPTIBLE 1 (PBS1) in *Arabidopsis* [14]. The *Arabidopsis* RESISTANCE TO *P. SYRINGAE* PV MACULICOLA 1 (RPM1) INTERACTING PROTEIN 4 (RIN4) can also be damaged by bacterial effectors. AvrRpt2 is a cysteine protease auto-processed inside the host cell and cleavages RIN4 [15]. Some effectors can damage their chitinase targets. The serine protease SSEP1 (secretory Ser-protease 1) is an effector secreted by V. dahliae to cleave apoplastic class IV chitinase Chi28 (Chitinase 28) of cotton [16]. The basidiomycete Ustilago maydis excretes UmFly1 (Fungalysin 1), a metalloproteolytic enzyme that hydrolyze maize chitinase ZmChiA [17].

#### 3.1.2 RNAs and DNAs

Ribonuclease (RNase) effectors may degrade host RNAs. *Fusarium graminearum* is a devastating fungal pathogen found worldwide. During the early stages of its infection in soybean, *F. graminearum* secretes an RNase effector Fg12 (F. graminearum secretome 12) to degrade host RNAs [18]. V. dahliae secretes a RNase effector named VdRTX1, which enters plant nucleus to modulate host immunity [19]. Some effectors cause host DNA damages instead. CRN13 (Crinkler 13) effectors produced by the amphibian pathogenic chytrid fungus *Batrachochytrium dendrobatidis* (BdCRN13) and the pathogenic oomycete *Aphanomyces euteiches* (AeCRN13) can enter host cell nuclei, trigger aberrant cell development, and ultimately cause cell mortality. Both CRN13s trigger a DNA damage response in host by hydrolyzing plant nuclear DNA [20].

#### 3.1.3 Cell wall and membrane

Plant cells are protected by a layer of cell wall. To overcome it, pathogens secret effectors called cell wall degrading enzymes. For example, TaGH61A from *Thermoascus aurantiacus* belongs to the glycoside hydrolase GH61 family and facilitates plant cell wall degradation to promote infection [21]. Plasma membrane is also a target of effectors. NLP proteins constitute a superfamily found in plant pathogenic bacteria, fungi, and oomycetes. NLP toxins form complexes with their sphingolipid receptors, Glycosylinositol phosphorylceramides (GIPCs). The interactions result in conformational changes within the toxins, forming transient small pores in lipid membranes [22].

#### 3.1.4 Molecular compounds

Some Toll/interleukin-1 receptor (TIR) proteins contain a Nicotinamide adenine dinucleotide NAD(+) hydrolases (NADase) domain, connecting NAD(+)-derived metabolites to immune signaling. Pathogens may also damage host NAD(+) metabolism through effectors. In *P. syringae*, thirteen effectors possess NAD(+)-hydrolyzing enzyme family domain(s). Most *P. syringae* strains encode at least one NAD(+)-hydrolyzing effector. Among these effectors, HopBY displays structural similarity to both adenosine diphosphate ribose (ADPR) cyclase and TIR. HopBY proficiently catabolizes NAD(+) and processes it into 2′cADPR, a metabolite that augments bacterial pathogenesis [23]. Chorismate mutases constitute integral enzymes of the shikimate pathway responsible for the conversion of chorismate into prephenate. The fungus *U. maydis* exudes a chorismate mutase dubbed Cmu1. Upon being uptaken into vegetable cells, Cmu1 disseminates to adjacent cells and commandeers chorismate metabolism [24]. Two effectors PsIsc1 (Isochorismatases 1) and VdIsc1, secreted respectively by *P. sojae* and *V. dahlia*, constitute isochorismatases capable of neutralizing salicylate (SA)-dependent intrinsic resistance through catalysis of the SA precursor isochorismate into pyruvate and 2,3-dihydro-2,3-dihydroxybenzoate [25].

### 3.2 Stratagem 2: inhibition

Inhibition is a frequently used stratagem for effector actions. It means that an effector directly inhibits the functions of its targets, such as enzyme activities, nucleic acid binding and transcription, protein stability and solubility (Fig. 3).

#### 3.2.1 Inhibits host enzyme activity

An example of effectors inhibiting host enzyme activity is AvrAC_Xcc8004_ from the pathogenic bacterium *Xanthomonas campestris*. AvrAC is an uridylyl transferase that adds uridine 5′-monophosphate to the activation loop of immune signaling cytoplasmic kinases BOTRYTIS-INDUCED KINASE 1 (BIK1) and RPM1-INDUCED PROTEIN KINASE (RIPK). Concealment of these sites reduces kinase activity and consequently inhibits downstream signaling [26]. Fungal effectors also show direct enzyme inhibiting activity. For example, NECROSIS-INDUCING SECRETED PROTEIN 1 (NIS1) is another BIK1 kinase activity-inhibiting effector, with two homologs CoNIS1 and MoNIS1 found in Colletotrichum orbiculare and *Magnaporthe* oryzae, respectively [27]. Other types of enzymes important for defense activation and also targeted by effectors. FKBP15–2 is a plant peptidyl-prolyl cis-trans isomerase (PPIase) and positively regulates plant immunity. The P. capsici effector Avr3a12 interacts with FKBP15–2 and inhibits its PPIase activity in vitro [28].

#### 3.2.2 Inhibits nucleic acid binding or transcription activity

Host transcription factors (TFs) and DNA binding proteins are also common inhibition targets of effectors. *Ascochyta rabiei* secretes an effector protein named PEXEL-like Effector Candidate 25 (ArPEC25), which targets the Lin11/Isl1/Mec3 (LIM) TF CaβLIM1a and obstructs its DNA-binding ability, culminating in negative regulation of the phenylpropanoid pathway and consequent hindrance of lignin production [29]. The vascular fungus *V. dahliae* infects plant roots to cause *Verticillium* wilt. Its effector small cysteine-containing protein 41 (VdSCP41) targets two *Arabidopsis* master immune regulators Systemic Acquired Resistance (SAR) Deficient 1 (SARD1) and CAM-BINDING PROTEIN 60-LIKE G (CBP60G). VdSCP41 binds the C-terminal portion of CBP60G to inhibit its TF function [30].

#### 3.2.3 Inhibits host protein stability

There are many examples of effectors inhibiting the stability of host targets. *Xanthomonas* outer protein D (XopD) is secreted by *Xanthomonas euvesicatoria*, the causal pathogen of bacterial spot disease in crops. It obtains a SUMO protease domain that directly binds to ETHYLENE RESPONSIVE ELEMENT BINDING FACTOR 4 (SlERF4) of tomato. XopD colocalizes with SlERF4 in subnuclear foci and destabilize it by catalyzing SUMO1-mediated hydrolysis from lysine 53 to suppress ET (Ethylene) production [31]. *P. syringae* produces the HopZ1a effector to promote the degradation of HopZ1-interacting JA-ZIM-DOMAIN proteins (JAZs) and activate JA pathway during infection [32]. Effectors from filamentous pathogens may also destabilize their targets. The CRN78 effector from *P. sojae* phosphorylates a soybean aquaporin, the plasma membrane intrinsic protein 2–13 (GmPIP2–13), to facilitate its degradation and promote infection [33].

#### 3.2.4 Inhibits host protein solubility

Interestingly, particular effectors decrease the solubility of their targets. A T3E XopR secreted by *X. campestris* undergoes liquid-liquid phase separation (LLPS) through intrinsically disordered regions (IDRs) and prevents *Arabidopsis* actin cytoskeleton from solubilizing. XopR and actin-binding proteins are assembled into a macromolecular complex at the cell cortex. By regulating the physico-chemical properties of XopR-complex coacervates, XopR progressively tampers with multiple stages of actin assembly, including filamentous actin (F-actin) crosslinking, actin depolymerization, and encompassing formin-mediated nucleation [34].

### 3.3 Stratagem 3: hijacking

An effector may hijack its targets by preventing interaction or relocalization (Fig. 3).

#### 3.3.1 Prevent interaction

Autophagy is a conserved eukaryotic degradation process involved in turnover of cellular components and biotic/abiotic stress response. Such process can be hijacked by phytopathogenic effectors. For example, Apple AUTOPHAGY 8i (MdATG8i) induces autophagy and resistance in response to *Valsa Mali* (Vm). During Vm infection, MdATG8i interacts with a plastid elongation factor Tu (MdEF-Tu) and degrades it through the autophagy pathway. Vm secretes Vm1G-1794, which competitively binds to MdATG8i and prevents the interaction between MdEF-Tu and MdATG8i to stabilize MdEF-Tu [35]. The effector Tin2 from U. maydis can mask the ubiquitination sites of maize ZmTTK1, thereby stabilizing this active kinase and upregulating genes responsible for anthocyanin biosynthesis [36]. Osp24 is a cytoplasmic effector secreted by F. graminearum. It can induce the degradation of wheat SNF1-related kinase TaSnRK1α by competing with wheat *Fusarium* Resistance Orphan Gene (TaFROG), which protects TaSnRK1α from the ubiquitin-26S proteasome [37].

Effectors can also hijack host DNAs. The *P. sojae* CRN effector PsCRN108 contains a putative DNA-binding motif and acts in plant cell nucleus to inhibit the expression of *Heat Shock Protein* (*HSP*) genes in *N. benthamiana, Arabidopsis* and soybean. PsCRN108 binds *HSP* promoters and disrupts the association between the heat shock element (HSE) and the plant heat shock TF, which activates *HSP* gene expression upon stress [38].

#### 3.3.2 Prevent relocalization

In potato, NAC TFs Targeted by *Phytophthora* (NTP) 1 and 2 are two ER membrane-located TFs. StNTP1 and StNTP2 proteins are released from the ER membrane following treatment with *P. infestans* culture filtrate to trigger plant immunity. They are targeted by *P. infestans* RXLR effector PITG_03192 (Pi03192) at the ER membrane. Pi03192 facilitates infection by preventing StNTP1 and StNTP2 relocalization from ER membrane to the nucleus [39]. Bremia lactucae RXLR effectors BLR05 and BLR09 also interact with the lettuce ER-associated tail-anchored NAC TF LsNAC069 and prevent its nuclear translocation [40]. Similarly, the Puccinia striiformis effector PstGSRE1 binds to the wheat ROS-associated TF TaLOL2 to inhibit its nuclear localization [41].

### 3.4 Stratagem 4: promotion

**Fig. 4:**
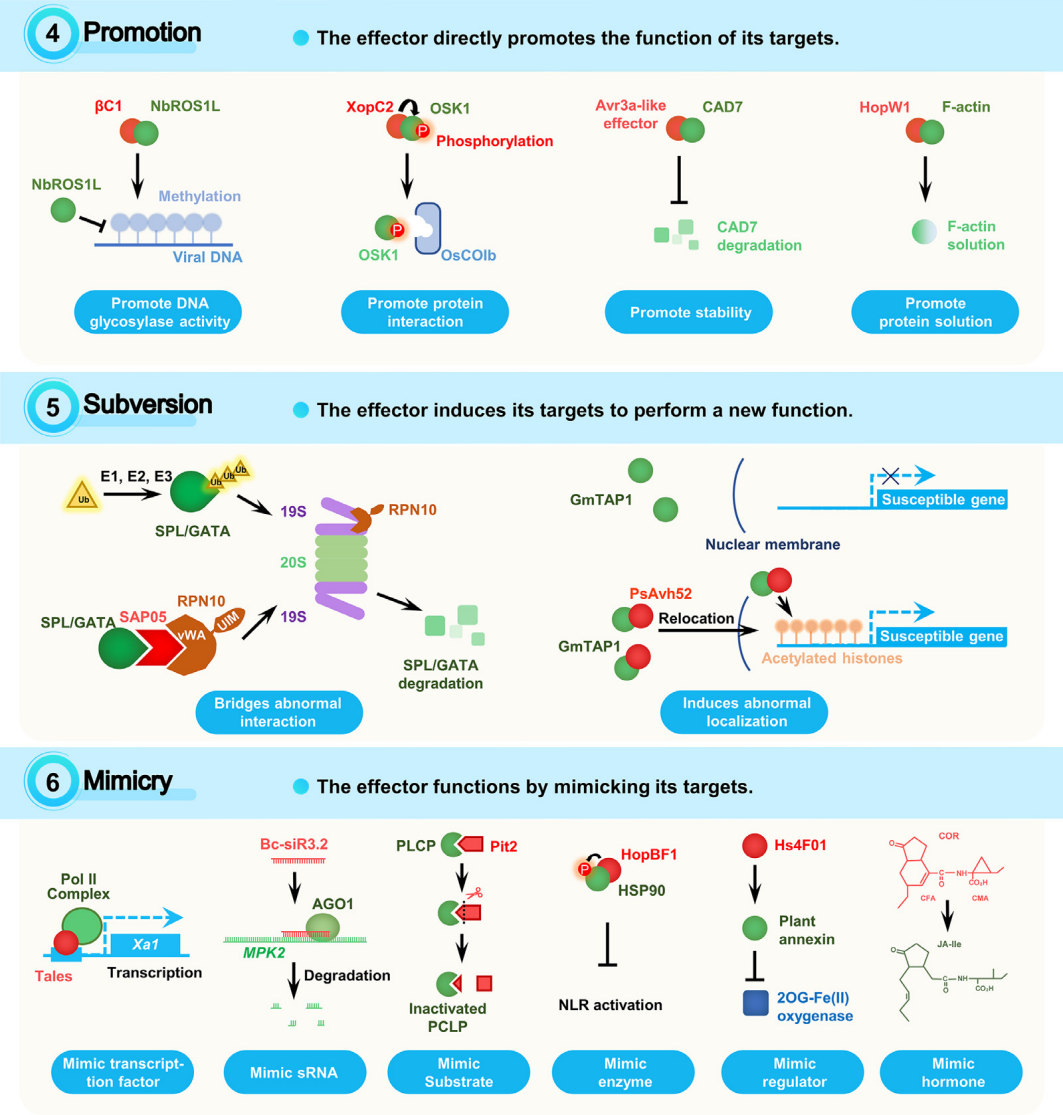
**Plant targets and effector stratagems: promotion, subversion, and mimicry**. The description of the indicated stratagems and the illustration of examples for these stratagems are shown. Effectors are shown in red. The targets of effectors are shown in green. Effector stratagems are written in above, with an upward arrow denoting increase and a downward arrow denoting decrease. The detailed target categories of the indicated effectors are written below.

Promotion means that an effector directly promotes the functions of its targets. The promotion effect can be on enzyme activity, nucleic acid binding capacity, protein interactions, target stability and solubility (Fig. 4).

#### 3.4.1 Promotes host enzyme activity

DNA methylation is an epigenetic mechanism that plays vital roles in transposon silencing and gene regulation pathways. This mechanism may also target viral DNAs, e.g., tomato yellow leaf curl China virus (TYLCCNV) and its satellite tomato yellow leaf curl China betasatellite (TYLCCNB). The βC1 effector encoded by TYLCCNB targets DEMETER (DME)/REPRESSOR OF SILENCING 1 (ROS1)-like DNA glycosylases in *Arabidopsis* and *Nicotiana benthamiana*. The interactions promote DNA glycosylase activity to downregulate viral DNA methylation and enhance TYLCCNB virulence [42]. In another example, AvrL567-A from Melampsora lini binds to the cytosolic cytokinin oxidase LuCKX1.1 of flax to elevate its catalytic activity [43].

#### 3.4.2 Promotes host protein interaction

The bacterial XopC2 effector family is widely distributed in *Legionella* species. XopC2 effectors are atypical kinases specifically phosphorylating Oryza S PHASE KINASE-ASSOCIATED PROTEIN 1 (SKP1)-like 1 (OSK1), a general receptor of the SKP1-Cullin-F-box ubiquitin ligase complex. Intriguingly, XopC2-mediated phosphorylation of OSK1 exclusively enhances the interaction between OSK1 and the rice JA receptor CORONATINE INSENSITIVE 1b (OsCOI1b), which promotes the ubiquitination and degradation of OsCOI1b and subsequently increase plant disease susceptibility by inhibiting stomatal immunity [44].

#### 3.4.3 Promotes host protein stability

Avr3a-like family effectors, which are constituted by homologs of *P. infestans* effector PiAvr3a, are a group of RXLRs widely found in diverse clades of *Phytophthora* species. Several Avr3a-like effectors can target and stabilize plant cinnamyl alcohol dehydrogenase 7 (CAD7) subfamily proteins, which are negative regulators of plant immunity [45]. The P. sojae effector PsAvh262 is an inhibitor of host endoplasmic reticulum (ER) stress-mediated cell death by increasing the stability of binding immunoglobulin proteins (BiPs), which function in the unfolded protein response (UPR) [46]. The *P. capsici* effector RXLR207 stabilizes *Arabidopsis* binding partners of accelerated cell death 11 (ACD11) (BPA) family proteins and subsequently enhances accumulation of the negative plant immunity regulator ACD11. ACD11 accumulation facilitates *P. capsici* transition from biotrophic to necrotrophic stage [47].

#### 3.4.4 Promotes host protein solution

Some effectors also promote the solubility of their targets. HopW1 is a T3E that increases *P. syringae* growth on *Arabidopsis*. When delivered into plant cells, HopW1 forms complexes with host actin to interfere the integrity of plant cytoskeleton. Its C-terminal region can decrease the length of actin filaments and therefore promote F-actin solubility [48]. By this way, HopW1 inhibits endocytosis, which is an actin-dependent process. The inhibition prevents transportation of certain proteins to vacuoles and finally promotes *P. syringae* colonization.

### 3.5 Stratagem 5: subversion

Subversion means that an effector induces its target to perform a new function (Fig. 4). Effectors using such stratagem often bridge abnormal interaction or induce abnormal localization.

#### 3.5.1 Bridges abnormal interaction

The *P. infestans* effector PexRD54 can subvert host autophagy mechanism by directing plant cells to form autophagic vesicles that *P. infestans* can then possibly use to feed on or to destroy antimicrobial components. PexRD54 bridges the cellular vesicle cargo protein and the autophagic structural protein surrounding the parasite, which directs the vesicles to the feeding sites of *P. infestans* for nutrient uptake [49]. The Arabidopsis TEOSINTE BRANCHED1/CYCLOIDEA/PROLIFERATING CELL FACTOR1 (TCP) 14 TF regulates plant immunity by transcriptionally repressing JA signaling cascade. TCP14 is the converged manipulation target of independently evolved ascomycete, eubacteria, and oomycete effectors. T3E HopBB1 from *P. syringae* connects TCP14 to JAZ3, the JA signaling repressor, and subsequently integrates it into the SCF^COI1^ degradation complex [50]. The insect-vectored phytopathogenic phytoplasmas secret SAP05 protein effectors that mediate concurrent degradation of plant GATA and SPOROCYTELESS (SPL) TFs via subverting the plant ubiquitin receptor RPN10. SAP05 bridges the TFs with RPN10 and induces their degradation in a ubiquitin-independent manner [51].

#### 3.5.2 Induces abnormal localization

Pi04314 is a RXLR effector of *P. infestans*. Ectopic expression of Pi04314 in plants enhances pathogen colonization on leaves. Pi04314 enters host nucleus and interferes the activation of SA/JA responsive genes. It targets three phosphatase 1 catalytic (PP1c) isoforms in plants, resulting in their relocalization from the nucleolus to the nucleoplasm to promote plant susceptibility [52]. Similarly, the P. sojae effector PsAvh52 translocates the *Glycine* max transacetylase GmTAP1 from the cytoplasm to the nucleus, where GmTAP1 acetylates histones H2A and H3 to facilitate P. sojae colonization [53].

### 3.6 Stratagem 6: mimicry

Effectors may function by mimicking their targets, including TFs, sRNAs, substrates, enzymes, regulators, and phytohormones (Fig. 4).

#### 3.6.1 Mimic TFs

The *Hyaloperonospora* arabidopsidis secretes an effector called HaRxL21, which contains a C-terminal EAR module that mimics host recruitment of the plant transcriptional co-repressor TOPLESS (TPL). By targeting TPL, HaRxL21 suppresses plant resistance to H. arabidopsidis [54].

#### 3.6.2 Mimic sRNAs

Some transkingdom transported small RNA-type effectors of the gray mold fungus *Botrytis cinerea* (Bc-sRNAs) can mimic host sRNAs and bind to host RNA interference (RNAi) machinery Argonaute 1 (AGO1) to selectively silence immunity genes in tomato and *Arabidopsis*, resulting in increased host susceptibility and facilitated *B. cinerea* infection [6].

#### 3.6.3 Mimic substrates

*U. maydis*-secreted effector protein Pit2 is an inhibitor of apoplastic papain-like cysteine proteases (PLCPs). Proteolytic cleavage of Pit2 by PLCPs liberates the embedded inhibitor peptide, which in turn inhibits PLCP activity to suppress host immune responses. Importantly, The core virulent motif of Pit2 is widely distributed in multiple plant-associated bacteria and fungi, indicating that they are a group of conserved microbial protease inhibitors [55].

#### 3.6.4 Mimic enzymes

Bacterial HopBF1-family effectors possess a kinase domain targeting eukaryotic HSP90. HopBF1 adopts a minimal protein kinase fold that mimics the endogenous client of HSP90 in hosts. As a result, HopBF1 phosphorylates HSP90, which completely blocks its ATPase activity and prevents the activation of plant immune receptors that trigger hypersensitive response (HR). Phosphorylation of HSP90 by HopBF1 is sufficient to cause severe symptoms in *P. syringae*-infected plants [56]. SA promotes the interaction between *Arabidopsis* NONEXPRESSER OF PR GENES 1 (NPR1) and the *P. syringae* T3E AvrPtoB, which leads to host 26S proteasome-mediated degradation of NPR1 through AvrPtoB's E3 ligase activity. In this case, AvrPtoB mimics the E3 ligase, promotes degradation of NPR1, and represses NPR1-mediated SA pathway, thereby suppressing plant immunity [57].

#### 3.6.5 Mimic regulators

Hs4F01 and its homologs are annexin-like effectors produced by the soybean root-parasitic cyst nematodes (*Heterodera* spp.). Hs4F01 shares high amino acid sequence similarity with *Arabidopsis* ANNEXIN 1. it contains a predicted signal peptide (SP) for secretion that is specifically identified in *Heterodera* nematode annexins. Hs4F01 also harbors four conserved domains typically found in the annexin family of phospholipid and calcium binding proteins. Hs4F01 targets an *Arabidopsis* oxidoreductase, which is demonstrated to promote plant susceptibility to oomycete pathogens. The secreted Hs4F01 may enter soybean root cells and mimic plant annexin function during parasitism [58].

### 3.7 Stratagem 7: reprogramming

**Fig. 5:**
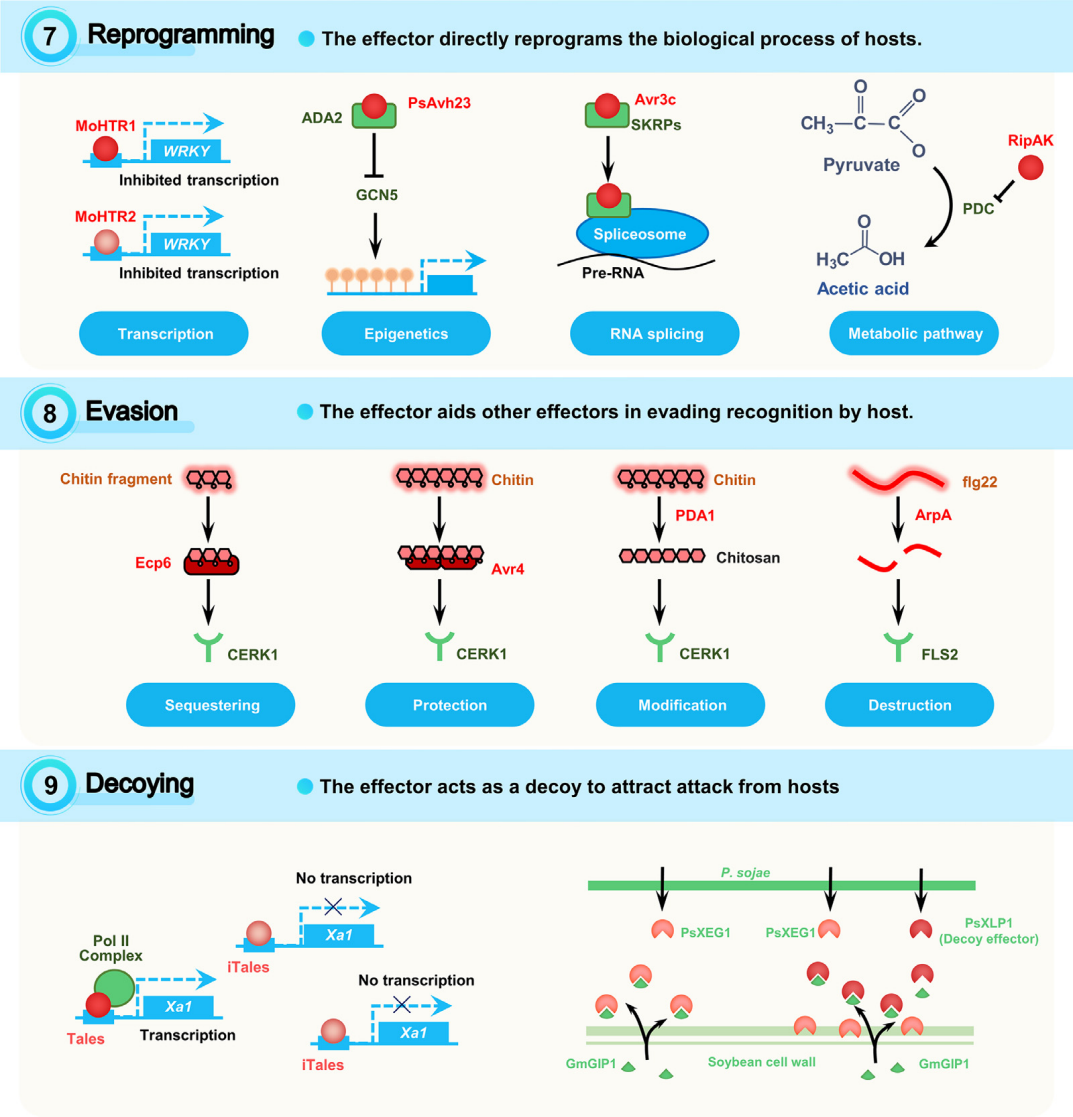
**Plant targets and effector stratagems: reprogramming, evasion, and decoying**. The description of the indicated stratagems and the illustration of examples for these stratagems are shown. Effectors are shown in red. The targets of effectors are shown in green. Effector stratagems are written in above, with an upward arrow denoting increase and a downward arrow denoting decrease. The detailed target categories of the indicated effectors are written below.

Effectors may directly induce reprogramming of host biological process (Fig. 5), such as transcription, epigenetics, RNA splicing and metabolic pathways.

#### 3.7.1 Reprogramming of transcription

Two *M. oryzae* nuclear effectors, MoHTR1 and MoHTR2, can translocate into nuclei not only in the cells initially penetrated by hyphae but also the surrounding cells. They then reprogram the expression of immunity-related genes by targeting targeted DNA elements [59].

#### 3.7.2 Epigenetic reprogramming

The cytoplasmic effector PsAvh23 produced by *P. sojae* modulates the histone acetyltransferase (HAT) activity of the SPT-ADA-GCN5 acetyltransferase (SAGA) complex in host plants. PsAvh23 disrupts SAGA assembly by binding to ADA2 and interfering its association with the catalytic subunit GCN5, which leads to the suppression of ADA2/GCN5-mediated histone H3K9 acetylation. This epigenetic reprogramming increases plant susceptibility to *P. sojae* [60].

#### 3.7.3 Reprogramming of RNA splicing

PsAvr3c, an effector from *P. sojae*, physically targets and stabilizes soybean serine/lysine/arginine-rich proteins (GmSKRPs), which are negative regulators of plant immunity and is one of the components of plant spliceosome. RNA-seq analysis indicates that PsAvr3c and GmSKRP1 affect the pre-mRNA alternative splicing of more than 400 soybean genes, including those related to defense [61]. Similarly, the effector Pst_A23 of P. striiformis also regulates alternative splicing of host resistance-related genes by targets splice site of specific pre-mRNA, thereby suppressing host defense responses and promoting infection [62].

#### 3.7.4 Reprogramming of metabolic pathways

The metabolic pathway governed by pyruvate decarboxylases (PDCs) is involved in plant resistance to bacterial wilt caused by *R. solanacearum*. Plant tolerance to the disease can be enhanced by treatment with either the product (acetic acid) or the substrate (pyruvic acid) of the PDC pathway. The *R. solanacearum* secreted effector protein RipAK can target PDCs to inhibit their self-interaction and enzymatic activity [63]. As another example, the fungus *M. oryzae* produces antibiotic biosynthesis monooxygenase (Abm) to change natural JA into hydroxylated JA (12OH-JA). Removing Abm (*Δabm*) from *M. oryzae* leads to upregulation of methyl JA (MeJA), triggering plant defense and blocking the spread of infection. Adding external 12OH-JA significantly reduces the defenses triggered by *Δabm* in rice. Notably, Abm itself is released after infection and likely converts host JA into 12OH-JA to help colonization [64].

### 3.8 Stratagem 8: evasion

Evasion refers to the observation that an effector aids other effectors in escaping host recognition via sequestering, protection, destruction, or modification (Fig. 5).

#### 3.8.1 Sequestering

The leaf mold fungus *Cladosporium fulvum* secretes LysM domain-containing effector Ecp6 to perturb chitin-triggered host immunity. During infection, Ecp6 sequesters chitin oligosaccharides derived from *C. fulvum* hyphae cell walls to avoid elicitating host defense response [65]. Furthermore, the M. oryzae effector MoAa91 enters the apoplast space to compete with the rice immune receptor chitin-elicitor binding protein (CEBiP). MoAa91 binds chitin and chitin oligosaccharides, thus inhibiting chitin-induced plant immune response [66]. MoChi1/MoChia1 is an extracellular chitinase effector of M. oryzae that competes with jacalin-related rice mannose-binding lectin 1 (OsMBL1) for chitin binding to inhibit chitin-triggered host defense [67].

#### 3.8.2 Protection

*C. fulvum* releases Avr4, a lectin-like effector with chitin affinity. Avr4 incorporates a chitin-binding motif (CBM14) that is prevalent among eukaryotes. For *C. fulvum* and other fungi such as *Fusarium solani* f. sp. Phaseoli and *Trichoderma viride*, Avr4 safeguards them from hydrolytic dismantling by host chitinases. [68]. The plant xylem pathogen Verticillium nonalfalfae secretes a CBM18 domain-containing effector VnaChtBP. VnaChtBP binds chitin oligomers to protect the fungal mycelium from degradation by chitinase [69].

#### 3.8.3 Modification

The N-acetyl group of fungal chitin oligomers is required for the perception by plant lysine motif (LysM)-containing receptor to trigger chitin-induced immunity. Adapted soil-borne fungi from the *Fusarium* and *Verticillium* genera secrete polysaccharide deacetylase (PDA) effectors to directly deacetylate chitin oligomers and prevent them from being recognized by plants [70].

#### 3.8.4 Destruction

Bacterial flagellin (FLG) proteins strongly activate intrinsic immune reactions in mammals and plants. The opportunistic phytopathogen *Pseudomonas aeruginosa* secretes an alkaline protease like effector AprA, which can cleavage FLG monomers to evade host detection [71]. Effectors with chitinase activity (EWCAs) are apoplastic effectors from fungal phytopathogens that hydrolyze chitin oligomers. They also emit various effectors incorporating lysin motifs to sequester chitin oligomers and stop them from linking with host sensors. For example, PEC5191 (Podosphaera effector candidates) from *P. xanthii* binds to chitin and suppress chitin-triggered immunity [72].

### 3.9 Stratagem 9: decoying

The decoying model means that the effector acts as a decoy to attract attack from hosts (Fig. 5), with only two cases found to date.

The *Xanthomonas* bacteria possess transcription activator-like effectors (TALEs) that activate the expression of rice susceptibility genes, which are recognized by rice nucleotide-binding leucine-rich repeat (NB-LRR) protein Xa1 through a decoy mechanism. However, some *X. oryzae* isolates use truncated TALEs (interfering TALEs; iTALEs) to defeat Xa1-mediated resistance. Compared to TALEs, iTALEs do not contain the transcription activation domain but retain DNA binding motifs, thereby inhibiting the expression of *Xa1* [73].

PsXEG1 is an apoplastic xyloglucan-specific endoglucanase effector secreted by *P. sojae*. Soybean glucanase inhibitor protein GmGIP1 can bind PsXEG1 to suppress its virulent function. *P. sojae* counteracts GmGIP1 by secreting a PsXEG1-like protein PsXLP1. PsXLP1 has no endoglucanase activity but interacts with GmGIP1 in higher affinity than that of PsXEG1. PsXLP1-GmGIP1 interaction frees PsXEG1 to promote *P. sojae* colonization [74].

### 3.10 Stratagem 10: adaption

**Fig. 6:**
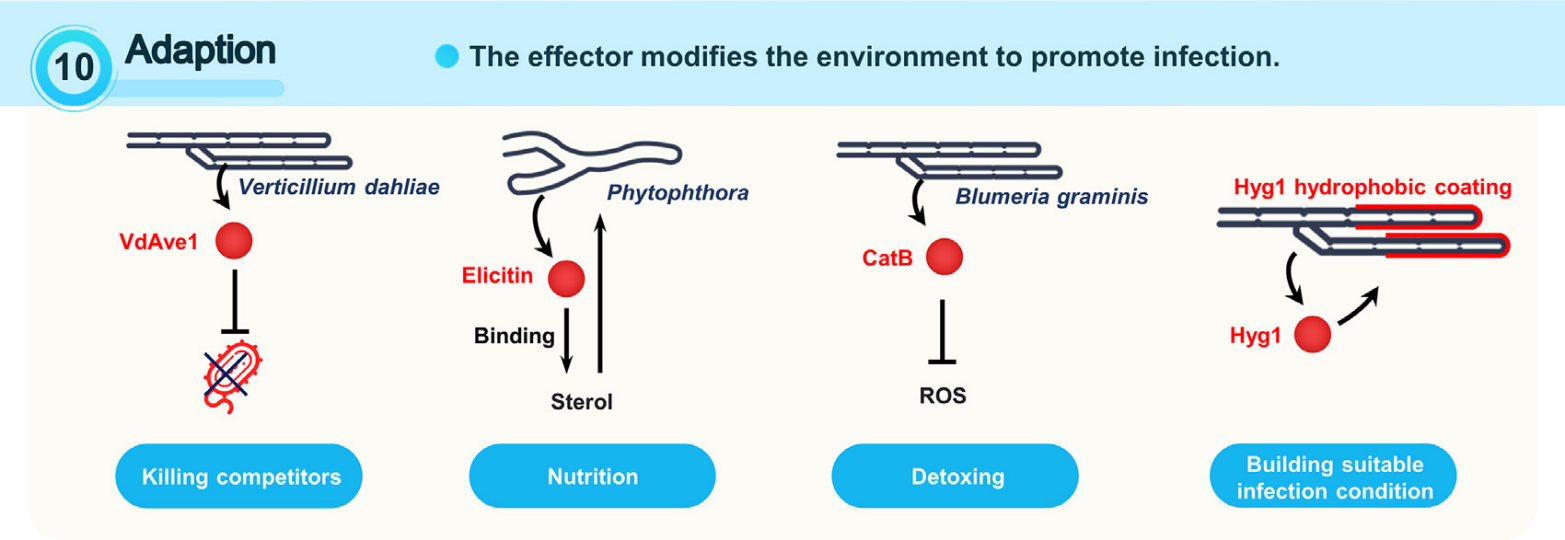
**Plant targets and effector stratagems: adaption**. The description of the indicated stratagems and the illustration of examples for these stratagems are shown. Effectors are shown in red. The targets of effectors are shown in green. Effector stratagems are written in above, with an upward arrow denoting increase and a downward arrow denoting decrease. The detailed target categories of the indicated effectors are written below.

In the adaption stratagem, the effectors modify the environment to promote infection, including killing competitors, nutrition achievement, detoxing, and building suitable infection conditions (Fig. 6).

#### 3.10.1 Killing competitors

Plants actively shape their microbiomes to suppress diseases. In turn, pathogens also evolve to manipulate host microbiomes towards their own advantages. The *V. dahliae* virulence effector VdAve1 manipulates tomato and cotton microbiomes by suppressing antagonistic bacteria. VdAve1 is a newly identified antimicrobial effector also from *V. dahliae* [12].

#### 3.10.2 Nutrition achievement

*Phytophthora* oomycetes cannot synthesize sterol, a key component of the plasma membrane and many signaling pathways. As sterol-heterotroph species, they secrete elicitins and effector proteins to trap and transfer sterols from host plasma membranes [75].

#### 3.10.3 Detoxing

The catalase effector from the barley fungal pathogen Bgh accumulates at the host-pathogen interface, which is consistent with the H_2_O_2_ clearing areas at the fungal invasion sites. These observations indicate that Bgh catalase activity may facilitate pathogenicity by detoxing H_2_O_2_ [13]. CfTom1 is a GH10 effector from the leaf mold C. fulvum and is required for C. fulvum to detoxify host-derived α-tomatine [76].

#### 3.10.4 Building suitable infection conditions

Some effectors promote mycelium and spore infestation by building a suitable environment between pathogens and host plants. Hydrophobins (HPs) are small proteins released by fungi. They spontaneously form single-layer membranes at the hydrophilic–hydrophobic interface. They provide a water-avoiding protein coating for fungal mycelium and spores, and helps spores spread and fungal mycelium grow into the air as they escape from wet areas [77]. In addition, the M. oryzae extracellular matrix protein EMP1 plays a role similar as HPs. *EMP1* knockout significantly suppresses pathogenicity and appressorium formation, but has no impact on sporulation or mycelium growth [78].

## 4 Concluding remarks and future perspectives

In conclusion, while a substantial number of effectors have been identified, their modes of action are mostly within the ten stratagems described above. However, there are also other possibilities outside the ten stratagems as novel effectors are continuously discovered. Furthermore, the rapid evolution of protein effectors and their functions are likely to generate novel modes of action beyond the ten stratagems. Despite that our knowledge about effector functions get boosted in the past 20 years, there are some emerging fields that need more attention and research efforts.

### 4.1 Effector cooperation

Effector numbers vary greatly in different pathogen species, ranging from as little as digits to hundreds per species. However, most effector proteins are only studied individually to date, which cannot reflect the broader context at the effectome level [79]. Effectome functions are usually not a simple sum of the individual effector functions. For example, HopAD1 Complementation DC3000Δ36E lines elicit immunity-associated cell death in *N. benthamiana*. Further complementation of AvrPtoB suppresses cell death, indicating an interplay between HopAD1 and AvrPtoB [80]. Recently, an interestingly work shows that bacterial virulence can be achieved through collective actions of cooperative effectors [81]. These findings highlight the importance of effector cooperation and should be paid more attention in the future.

### 4.2 Transcriptional regulation and epigenetic regulation of effectors

As effectors are key determinants of infection, they are precisely controlled by transcriptional regulation and epigenetic regulation in pathogens, and the abundance of pathogenic effectors during pathogen-host interaction is thus quite dynamic [82,83]. For example, RXLR207 from *P. capsici* is only induced during the biotrophic-necrotrophic transition stage and facilitates the transition by promoting host defense and cell death [47]. In contrast, ectopically expressing RXLR207 in the host will consistently activate host immunity and inhibit *P. capsici* colonization [47]. In *P. sojae*-soybean interaction, host cell death-inducing effectors are also likely induced during biotrophic-necrotrophic transition stages [84]. Similarly, such phenomenon also widely exists in fungal pathogens [83]. One should note the effector expression pattern and consider it in experimental design, or a wrong phenotype could be found. A study directly highlights such an opinion: engineering the PsAvr3b promoter sequence by substitution with different promoter sequences (constitutive expression, early expression or later expression) impacts the consequence of plant-*Phytophthora* interaction [85].

### 4.3 Effectors in post-AlphaFold era

Effector 3D structures are useful to show how they are recognized by plant receptors, or reveal the visualized conditions of how effectors attack their host targets [86]. Protein structures were historically analyzed using the combination of experimental and *in silico* methods. With the recent AlphaFold revolution in 2020s, pure computational methods can be used to accurately predict the structures of most ordered proteins as well as numerous protein–protein interactions [87]. Nowadays, the structures of many effector protein sequences have been predicted with AlphaFold and stored in UniProt.

Predicted effector structures provide useful clues for effector identification and evolutionary analysis. *M. oryzae* effectors AVR-Pia and AVR1-CO39 share low protein sequence identity, but they both have a close six-β-sandwich structure. Further structure-informed pattern searches identify additional novel effector candidates with similar structures in a vast spectrum of ascomycete phytopathogens [88]. Recently, a large-scale effector 3D structure analysis using AlphaFold 2 was conducted on secreted proteins from 14 crucial agricultural fungal pathogens, one oomycete, and six non-pathogenic fungi [89]. The authors find uniquely expanded, sequence-unrelated, but structurally similar effector (SUSS) families in pathogens investigated. They also demonstrate that SUSS effector evolution is shaped by divergent evolution events [89]. These findings highlight a novel effector identification method based on 3D structure, which is useful for digging out effectors with no conserved motifs or domain in secondary structures. Furthermore, 3D structures contain information about how a specific effector domain interacts with the target protein and provide potential engineered target to edit target genes to evade effector inhibition. For example, a cyst nematode virulence effector binds and inhibits the helper NLR (nucleotide binding and leucine-rich repeat) protein NRC2 (NLRs required for cell death 2). According to 3D structure, an amino acid polymorphism at the binding interface between NRC2 and the inhibitor is sufficient for this helper NLR to evade immune suppression, thereby restoring the activity of multiple disease resistance genes [90]. Overall, the structure-based effector study is a nonnegligible research direction in the future.

### 4.4 Learning from effectors to compete with them

Effector studies help us find ways to compete with them and recapture the agricultural lost from pathogens. Here, we focus on three promising effector-interfering strategies that provide prolonged resistance to pathogens with low possibility of being overcome by the quick effector evolution.

#### 4.4.1 Targeting effector delivery pathways

Cytoplasmic effectors have to enter host cells to function. Interfering this pathway may greatly reduce pathogen pathogenicity. For example, RXLR effectors enter host cells through the association with external lipid phosphatidylinositol 3-phosphate (PI3P) [91]. Furthermore, oomycete pathogens can produce abundant PI3P that direct binds RXLRs to facilitate infection [92]. Our lab developed a novel strategy to control oomycete disease by decreasing pathogen-derived PI3P through expressing *Arabidopsis* phosphatidylinositol-4-phosphate 5-kinase 1 (AtPIP5K1) and translocating the kinase to plant apoplast to metabolize PI3P to PI(3,4)P(2). Transgenic potato and soybean crops adopting this strategy show significantly enhanced resistance to various *P. infestans* and *P. sojae* isolates [93]. We also fuse anti-microbial proteins with the PI3P-binding domain FYVE to guide them to pathogen hyphae with high efficiency, which greatly reduces *Phytophthora* colonization [94].

#### 4.4.2 Knockout effector targets and complement with non-host substitutes

Many effector functions depend on their subversion of host targets. Knockout of these targets might improve host resistance. For example, P. sojae PsAvh52 targets soybean transacetylase GmTAP1 to promote early infection. CRISPR/Cas9-mediated knockout of GmTAP1 significantly increases soybean resistance to *P. sojae* [85]. However, knocking out host genes might induce unexpected side effects. Recently, two interesting works have provided a potential solution for the issue. Phytoplasma SAP05 binds host RPN10 to promote infection. A two-amino-acid substitution within plant RPN10 generates a SAP05-resistant but still functional variant [51], which is expected to have reduced side effects. Although pathogen effectors are associated with their host targets, they hardly bind to their target homologs in non-hosts. Complementation using target homologs from non-hosts may significantly improve plant resistance with no obvious side effects [95]. Thus, knocking-out effector-targeted genes and complementation with their non-host homologs might be a good strategy to dysfunction pathogen effectors.

In conclusion, as the arms race between pathogens and plants is an endless evolutionary process, our battle with pathogen effectors also needs constantly evolving technologies to stay at least one step ahead of pathogens.

## Declaration of competing interest

The authors declare that they have no conflicts of interest in this work.
